# Evaluating patient experience in maternity services using a Bayesian belief network model

**DOI:** 10.1371/journal.pone.0318612

**Published:** 2025-02-20

**Authors:** Abrar Abdulhakim Ahmed Munassar, Mecit Can Emre Simsekler, Ahmed Alaaeldin Saad, Abroon Qazi, Mohammed A. Omar

**Affiliations:** 1 Department of Management Science & Engineering, Khalifa University of Science & Technology, Abu Dhabi, United Arab Emirates; 2 School of Business Administration, American University of Sharjah, Sharjah, United Arab Emirates; University of Babylon, IRAQ

## Abstract

Pregnancy and childbirth are commonly seen as positive experiences, but they can also pose distinct challenges and risks, especially when care is insufficient. This study investigates the factors influencing maternity patient experience by exploring the complex interactions among these factors. Using data from the 2021 maternity patient survey by the National Health Services (NHS) in England, we implemented a Bayesian Belief Network (BBN) to model these interactions. Three structural learning models were created, namely Bayesian Search (BS), Peter-Clark (PC), and Greedy Thick Thinning (GTT). Further, sensitivity analysis was conducted to quantify interactions among the influencing factors and identify the most influential factor affecting the outcome. The results underscore the importance of recognizing the interdependencies among the eight key domains of the survey, which collectively shape maternity care experiences. These factors include *the start of care in pregnancy, antenatal check-ups, care during pregnancy, labour and birth, staff caring, care in the hospital, feeding the baby, and care after birth*. These findings can guide healthcare managers and decision-makers in developing proactive strategies to mitigate factors impacting maternity patient experiences. Ultimately, this study contributes to the ongoing efforts to enhance the quality of maternity care and improve outcomes for mothers and their infants.

## 1. Introduction

The birth of a child is a monumental occasion for many women, especially those starting their parenting journey. Therefore, happy memories from this time are important as they assist women emotionally and strengthen them in their parental role. Conversely, a challenging or poor pregnancy experience considerably raises the likelihood of adverse health outcomes for the mother and her baby potentially leading to both physical and psychological complications [1]. Maternity care extends beyond medical treatment, including emotional support, information, and education [2]. Moreover, the fragmented nature of maternity makes it complicated care where it involves a wide range of services and healthcare professionals [3,4]. Given this complexity, successful outcomes for pregnant women and their newborns are dependent on maternity care that is accessible, inexpensive, and of high quality throughout the pregnancy, childbirth, and postnatal period [5].

Poor quality of care, influenced by multiple factors, can discourage women from seeking medical assistance, leading to preventable maternal deaths [6,7]. In 2015, an estimated 303,000 women worldwide lost their lives due to preventable complications related to pregnancy and childbirth [8], highlighting the need for maternity care quality improvements. These high maternal mortality rates indicate systemic challenges in service delivery, negatively affecting patient experience. To ensure high-quality maternity care and improve patient experience, it is essential to identify and address the various factors shaping these experiences and their interactions.

Healthcare practitioners, researchers and policymakers actively explore the factors affecting women’s satisfaction in maternity care [9]. For example, several studies addressed critical factors that may have contributed to the maternity patient experience [10–13]. However, significant gaps remain in the literature. Firstly, there is a lack of consensus on which factors are most influential, as various authors have different perspectives based on their observations. Secondly, different factors influencing maternity care experiences have been identified, but there is a neglect of the probabilistic interactions between these factors and how improving one of them will influence the others and the whole [14]. Moreover, based on the authors’ observations, there is an opportunity to explore additional factors with the assistance of experts. Accordingly, this study aims to fill these gaps and addresses the following research questions: (1) What are the primary factors that influence maternity patients’ experience throughout their journey? (2) How do these factors interact probabilistically to impact patients’ experience in maternity services? (3) How can these insights guide better decision-making to improve both patient experience and service quality?

To achieve this, a Bayesian Belief Network (BBN) was employed to model and explore the interactions between these factors, as BBNs have proven effective in decision-making across key areas such as clinical decision support [15]. This approach provides insights for policymakers and healthcare managers to design comprehensive, system-wide interventions that reflect the reality of interacting factors in maternity care, moving beyond the traditional siloed approach.

This paper is structured as follows: Section 2 provides an overview of both the maternity patient experience and the BBN literature. Section 3 outlines the methodology utilized. Section 4 presents the findings and their interpretations. Section 5 presents key findings, highlights the study’s implications, acknowledges its limitations, and suggests avenues for further research.

## 2. Literature review

### 2.1. Maternity patient experience

The concept of the patient experience is complex and interconnected, comprising several aspects and features [16]. Therefore, the term “patient experience” does not have a universally agreed-upon or established definition [17]. However, as per The Beryl Institute, the patient experience is characterized as “the sum of all interactions, shaped by an organization’s culture, that influence patient perceptions, across the continuum of care” [18]. In the context of maternity care, a study describes maternity patient experiences as their evaluation of care encounters within maternity services, which is subjective and changes throughout pregnancy, childbirth, and postpartum. The authors also emphasized that a woman’s needs, preferences, and expectations influence her perception of maternity care service [19]. Moreover, the WHO defines maternity patient experience along three components: effective communication, respect and dignity, and emotional support [20].

The World Health Organization’s (WHO) Global Strategy for Women’s, Children’s, and Adolescents’ Health (2016–2030) emphasizes the growing importance of women’s health globally [21]. This strategy emphasizes comprehensive, integrated healthcare services, including maternal care and quality healthcare throughout women’s lives. The WHO highlights the importance of women’s experiences in providing high-quality maternity care [22]. Investing in women’s health is crucial for lowering maternity and infant mortality, improving overall health outcomes, and boosting socioeconomic development, with high-quality care serving as a foundation for women’s empowerment worldwide [23]. In the past decade, there has been an increasing focus on gathering patient experience data in real-time to enhance care delivery [24] and the importance of survey instruments quantifying women’s experiences during maternity care has been acknowledged globally with many countries employing such instruments to guide policy and practice [4,25] (see Table 1). These surveys have provided valuable insights into different stages of maternity care, yet important gaps remain in understanding how these experiences interrelate throughout the entire maternity journey.

**Table 1:** Surveys for maternity patient experience evaluation.

Country	Survey Title	Purpose
Canada	Maternity Experiences Survey (MES)	Measure women’s experiences, behaviors, views, and knowledge throughout pregnancy, delivery, and postpartum [26].
Ireland	National Maternity Experience Survey (NMES)	Assess service patients’ experiences in maternity care [4].
United Kingdom (NHS)	Maternity Patient Experience Survey	Focuses on antenatal care, labor and birth, and postnatal care, as well as feeding and post-birth care [1].

International efforts have been focused on improving patient experience, particularly in maternity care. Many studies have been conducted to understand women’s satisfaction, expectations, and overall experiences, leading to the identification of various influencing factors [27–29]. Effective communication has been identified as a critical factor in shaping patient experience [30]. Additionally, involvement in decision-making [31,32], a sense of control [33], and the quality of relationships with caregivers have been found to play important roles [34,35].

Moreover, in response to the need for more comprehensive care model, various mother-friendly care models have been introduced, such as Women-Centred Care (WCC), Person-Centred Care (PCC), and Family-Centred Care (FCC) [36]. Women-Centred Care (WCC) is defined as delivering care that respects and responds to the preferences, needs, and values of women and families [37]. PCC and FCC have improved service accessibility and the overall quality of care [38,39].

Despite these advancements, significant challenges remain because most existing research focuses on isolated phases of maternity care, ignoring the interrelationships between these stages[40,41], This has resulted in the failure to address many hidden factors and the probabilistic interdependencies between these factors that affect maternity patient experience.

This study, unlike previous studies, aims to examine influential factors in the maternity patient experience as a holistic journey rather than discrete phases and uncover meaningful connections between these factors. By utilizing data from the NHS’s Maternity Patient Experience (MPE) survey and applying a data-driven Bayesian Belief Network (BBN), we seek to provide a comprehensive understanding of how various aspects of care interact to shape maternity experiences.

### 2.2. Bayesian belief network

Bayesian Belief Networks (BBNs) are powerful tools used for probabilistic modeling, particularly effective in fields that involve uncertainty and complex interrelationships [42]. BNNs are graphical representations of joint probability distributions (JPDs), presented as networks comprising nodes and edges [43]. They are especially useful in diagnosing complex problems, supporting healthcare professionals by revealing causal connections in uncertain environments [44]. Moreover, BBNs have been utilized to assess risks in medical information systems, demonstrating their value in addressing complex issues in healthcare settings [45].

**Fig 1:**
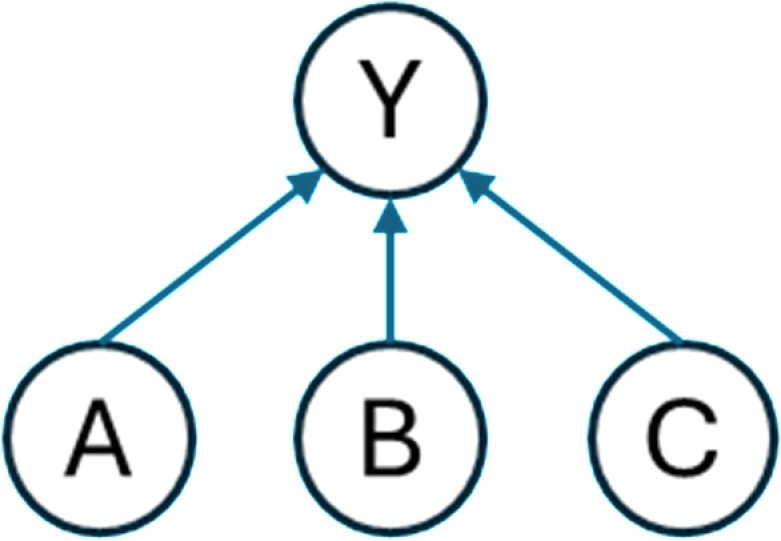
A simple Bayesian Belief Network representation.

A BBN consists of both qualitative and quantitative components. The qualitative component is represented by a directed acyclic graph (DAG), in which nodes represent system variables and directed arcs show dependencies or cause-effect relationships (Fig 1) [46–48].

The quantitative component involves conditional probability tables (CPTs), that represent the conditional relationships between nodes and their corresponding parent nodes, reflecting how likely particular outcomes are given certain conditions [43,49,50]. The joint probability distribution of n nodes is represented as Equation 1 [51]:


$$\text{P}\left({\text{x}}_{1},\ldots .,{\text{x}}_{\text{n}}\right)=\overset{\text{n}}{\underset{\text{i}=1}{\prod }}\text{P}\left({\text{x}}_{\text{i}}{\text{|Pa}}_{{\text{x}}_{\text{i}}}\right).$$ (1)

where $Pa_{x_{i}}$ represents the set of parent variables for each random variable $x_{i}$.

To better illustrate these relationships, Fig 1 provides an example of a simple BBN that depicts the interdependencies among four variables A, B, C, and Y. The directed arrows from A to Y, B to Y, and C to Y indicate that Y depends on all A, B, and C. In this reliance scheme, Y is referred to as the child node, while A, B, and C are referred to as the parent nodes.

It is worth mentioning that various tools and methods are used for informed decision-making. However, the existing literature regularly highlights the considerable usage of Bayesian networks, neural networks, and decision trees in the creation of medical expert systems and clinical decision support systems [52–54]. However, in this study, BBNs were chosen over alternative approaches due to its ability to generate and assess the impacts of various what-if scenarios, making it well-suited for decision-making in contexts involving risk and uncertainty unlike the other approaches [43,48]. Additionally, one key advantage of BBN is its high tolerance for missing data, as it can still generate reliable insights when some data points are unavailable. In contrast, ANN has a low tolerance for missing data, which can hinder its performance, and decision trees have a moderate tolerance for missing values [55]. These characteristics make BBN particularly advantageous for our study, as it allows us to model probabilistic dependencies and manage missing data effectively in the healthcare context. Furthermore, its use is justified because of its capacity to assess multi-state variables and update probability [16,56]. This means a BBN can improve its performance over time as it encounters more cases. BBNs are commonly accepted as an effective framework in healthcare [57]. Table 2 summarizes key studies that highlight the effectiveness of BBNs in various healthcare applications.

**Table 2:** Applications of Bayesian Belief Networks in healthcare.

Application area	Study highlights
Breast Cancer Survival [58]	Effectiveness of BBN in establishing interrelationships between key factors, determining conditional survival probabilities, and predicting survival outcomes.
Bone Sarcoma Survival Risk [59]	BBN used to develop a decision-support tool for customizing short-term survival risk for patients with bone sarcoma, aiding in risk-informed decisions.
Risk Analysis in Medical Settings [60]	BBN employed to predict likelihood of patient no-shows, integrated with expert insights to improve facility utilization, intervention strategies, and patient outcomes.
Safety Climate and Performance [61]	BBN utilized to analyze correlations between safety climate dimensions, safety performance, and occupational accidents in hospitals, identifying key safety factors.

Although BBN has been widely used in healthcare research, there is limited literature on its application to explore the interconnections and significance of factors influencing maternity patient experience [62,63]. This study aims to address this gap by utilizing BBNs to identify influential factors across the maternity care continuum, thereby providing insights into how experiences in one phase can impact perceptions and outcomes in subsequent stages. This approach offers a novel contribution by viewing maternity care as an interconnected journey rather than isolated phases.

While BBNs offer many advantages, such as high tolerance for missing data and effective scenario analysis, they do have limitations, including their inability to represent true feedback loops and dynamic time-based interactions [64]. This explains why the relationships may appear reversed, they reflect probabilistic dependencies, not true feedback loops, meaning experiences in later stages can influence perceptions of earlier phases. However, for analyzing maternity care as a set of probabilistic dependencies, these limitations are acceptable.

## 3. Methodology

### 3.1. Data acquisition and preparation

The dataset used in this research comprises trust-level Maternity Patient Satisfaction survey data from 2021 conducted by the National Health Services (NHS) in England [65]. The survey, initiated in 2007 as part of the NHS Patient Survey Programme (NPSP), evaluates women’s experiences of maternity care across England. Its primary goal is to provide NHS trusts with insights to drive targeted quality improvements, while also supporting the Care Quality Commission (CQC) and NHS England in performance assessments, regulatory evaluations, and frameworks.

The research team accessed this data in 2023 for research purposes and did not have access to information that could identify individual participants during or after data collection. As the survey was conducted during the third national COVID-19 lockdown, respondents experienced their pregnancy, delivery, and postnatal care during the pandemic, which likely influenced their perceptions and may have introduced response bias, complicating comparisons with previous years. Additionally, changes in health-seeking behavior during the pandemic, such as delays in care or decreased access to services, may have influenced respondent selection, introducing selection bias. This is based on the authors’ perceptions, as the NHS has not disclosed any biases during data collection. Nonetheless, these factors should be considered, as they may reflect challenges that may affect the results.

The dataset comprises 122 trust-level observations, each representing responses to 50 selected questions used to construct the BBN model. The survey is divided into sections that correspond to different stages of the maternity care experience, including the start of care during pregnancy, antenatal check-ups, care during pregnancy, labor and birth, staff caring, care in the hospital, feeding the baby, and care after birth. From the original 74 questions, 50 were selected by the NHS for inclusion in these eight sections. The selection and grouping of the questions into sections were made by the NHS as part of their survey design. The author used this predefined structure without modification. The section mean represents the average of all relevant questions within that section (see Table A1 in S1 Appendix for the complete list of questions and sections).

While the dataset has been used for performance evaluations, utilising our knowledge, no previous studies have explored the interdependencies of factors influencing maternity care experiences using this data.

### 3.2. Data preprocessing

After obtaining this dataset, the next stage includes thorough cleanup procedures that involve outlier removals coupled with handling missing observations. In the subsequent step, statistical analyses were executed, including computing the Cronbach alpha coefficient to evaluate the reliability of the survey questionnaire [66,67]. Additionally, descriptive analyses were conducted to gain insight into the nature of the data.

Statistics experts have disagreed on the ideal Cronbach’s alpha value. However, according to the literature review in the context of human dimensions research an alpha value between 0.50 and 0.95 is considered adequate [67].

Managing missing data is a common challenge in medical research [68,69], and several methods have been suggested to impute missing data [70]. In this study, mean imputation was employed to fill in missing values, as it is a straightforward and widely accepted method when the proportion of missing data is relatively low [71]. The proportion of missing data in the used dataset was relatively low, at 7%, which justified the use of this approach.

### 3.3. Data discretization

Data discretization was carried out in the third phase, as it is proven that BBNs can create improved models by discretizing continuous characteristics [55]. There are various types of discretization algorithms [72,73]. Most common approaches are equal width, equal frequency, and k-mean discretization [72]. An earlier study showed that the application of an equal-width discretization scheme with the Naive Bayes classification model resulted in improved accuracy [74]. This study has obtained a similar conclusion, as the equal-width scheme achieved the highest accuracy among the three tested algorithms. The initial step in determining the number of states for each variable was conducted using k-means clustering. The silhouette and elbow methods were used to determine the ideal number of clusters for each variable, resulting in some variables being clustered into two and others into three. Once these optimal cluster sizes were determined, we applied the same discretization scheme to the equal width and equal frequency methods, ensuring that all discretization techniques followed consistent logic. This process led to the creation of different states, such as **C0, C1, and C2**. These States refer to the discretized levels of the factors being analyzed. For example, in the context of the staff caring factor, state 1 might represent an acceptable level of care, while state 2 represents an excellent level of care. These states were used to categorize the responses.

### 3.4. BBN modelling

The BBN model was created to explore and capture the complex interdependencies between various factors influencing maternity patient experiences. To construct the model, the dataset was discretized using the equal-width approach and then imported into GeNIe software. Due to the absence of a predefined target variable, various structural learning algorithms were employed to model the intricate relationships among factors affecting patient experiences in maternity services, making it well-suited for uncovering hidden relationships within the dataset. Three structural learning algorithms were used, including Bayesian search (BS), Peter Clark (PC), and Greedy Thick Thinning (GTT), each applied to datasets with different discretization schemes (two-state, three-state, and mixed-state), resulting in the creation of nine BBN models each of which aimed to reveal the probabilistic interdependencies among the factors affecting maternity experiences.

### 3.5. Model validation

Following the structural learning phase, predictive modeling was implemented to validate the findings. Validation is conducted for the nine models to confirm that the BBN model’s structure was not only descriptive but also had predictive power, particularly for the most critical influencing factor. In BBN modeling, techniques such as confusion matrix, receiver operating characteristic (ROC) curves, k-fold cross-validation, have proven useful tools and measures [75]. GeNIe software supports three validation techniques: *K*-fold cross-validation, test-only, and leave-one-out. This study utilized k-fold cross-validation, where the data was split into k = 10 folds. In this process, the model is trained on k-1 folds and tested on the remaining fold. This is repeated for each fold, and the performance metrics are averaged to ensure the model generalizes well across different subsets of the data [76]. By using this approach, we avoided the risk of overfitting that can arise from fitting the model to a single training set, as the model was validated on different test sets across iterations. The choice of k = 10 is supported by literature, which identifies it as an optimal value for BBN models [77].

### 3.6. Sensitivity analysis

Sensitivity analysis helps identify critical factors and processes that contribute to the overall uncertainty within the model. It reveals how sensitive the results are to minor variations in the evidence values [78]. by using the built-in features in the GeNIe software, we were able to measure the impact of each variable on the model. This analysis enabled us to identify the most essential element and confirm that the model appropriately depicts the interactions between all the factors, which can assess the robustness of our decisions.

## 4. Results

### 4.1. Data analysis

The internal reliability of the eight sections of the original survey was evaluated after data cleaning and ensuring the absence of missing data. The eight sections demonstrated an acceptable internal consistency, with an alpha value falling between 0.50 and 0.95 which can be considered acceptable [67] (see Table 3).

**Table 3:** Internal reliability calculation for the original NHS survey.

The section	Cronbach’s alpha
S1: The start of the care in pregnancy	0.83
S2: Antenatal check-ups	0.75
S3: During the pregnancy	0.79
S4: The labour and birth	0.74
S5: Staff caring	0.92
S6: Care in the hospital	0.53
S7: Feeding the baby	0.70
S8: Care after birth	0.92

Following the confirmation of the internal reliability, descriptive analysis was conducted for all eight sections to gain insights into the nature of the data. Table 4 presents the data after mean imputation, which was applied at the section level to handle missing data. This ensures that the sample size remains consistent at 122 across all sections. It reveals a positively skewed distribution, largely because respondents tended to give high ratings across several aspects of maternity care. Moreover, the low standard deviation across sections indicates little variability in responses, reflecting consistently positive experiences. This reduced variability may affect the ability to distinguish between key factors in the BBN model.

**Table 4:** Descriptive summary of the original survey sections.

ID	Section name	Mean	Median	Std	Min	Max	Sample Size
S1	The start of the care during pregnancy	5.10	4.99	0.51	4.14	6.70	122
S2	Antenatal check-ups	7.95	7.93	0.36	6.93	8.86	122
S3	During the pregnancy	8.26	8.29	0.35	7.26	9.06	122
S4	The labour and birth	8.13	8.14	0.36	7.13	8.85	122
S5	Staff caring	8.35	8.37	0.31	7.48	8.91	122
S6	Care in the hospital	7.03	6.98	0.53	5.62	8.52	122
S7	Feeding the baby	8.28	8.29	0.32	7.36	8.90	122
S8	Care after birth	7.51	7.53	0.36	6.36	8.42	122

Table 5 illustrates the correlation matrix which was built to evaluate the level of interaction among the eight sections. All sections demonstrate a positive correlation, emphasizing the interconnectedness of maternity care, where early experiences shape later outcomes, highlighting the need for integrated, continuous care. However, the strongest correlation (0.80) between **Antenatal Check-Ups (S2) and During Pregnancy (S3)** indicates that adequate antenatal care leads to positive pregnancy experiences. Similarly, the strong correlation between Staff Caring (S5) and Labor and Birth (S4) (0.77) demonstrates the influence of interpersonal care on labor outcomes.

**Table 5:** The correlation matrix of the original survey sections.

	S1	S2	S3	S4	S5	S6	S7	S8
S1	1							
S2	0.63	1						
S3	0.61	0.80	1					
S4	0.49	0.50	0.63	1				
S5	0.41	0.43	0.61	0.77	1			
S6	0.33	0.34	0.43	0.50	0.57	1		
S7	0.32	0.43	0.60	0.50	0.61	0.65	1	
S8	0.42	0.66	0.67	0.50	0.57	0.50	0.61	1

Thereafter, the analysis focused on comparing different discretization methods within the BS model. The PC and GTT algorithms were excluded from this table, as the primary objective was to assess the impact of various discretization techniques specifically on the BS model, which served as the standard for comparison (see Table 6).

**Table 6:** Different discretization methods with prediction accuracy.

	Discretization method	Accuracy
Two states (Bayesian search)	Equal width	**0.70**
	Equal frequency	0.69
	K-mean	0.66
Three states (Bayesian search)	Equal width	**0.72**
	Equal frequency	0.64
	K-mean	0.61
Mixed state (Bayesian search)	Equal width	**0.77**
	Equal frequency	0.67
	K-mean	0.62

With different data schemes, the equal-width algorithm has achieved the highest accuracy among all the other approaches as highlighted in Table 6. Therefore, any further analysis was based on the **equal-width discretization** method.

### 4.2. Model performance and evaluation

While the primary focus of this study was the development of a BBN to model interactions between factors, we also conducted a follow-up analysis to evaluate the model’s predictive capabilities, specifically for the most critical factor identified. Based on the sensitivity analysis, the **staff caring** factor emerged as the most influential variable impacting patient experience. To assess the predictive performance of the model, we employed GeNIe’s built-in k-fold cross-validation feature across all nine implemented models. For each model, two key performance measures accuracy and Receiver Operating Characteristic curve (ROC) were calculated, and the highest-performing value for each metric is highlighted in bold (see Table 7).

**Table 7:** Performance metrics of various BBN models.

States	Algorithm	Accuracy	ROC
Two states	Bayesian search (BS)	0.70	0.74
	Peter-Clark (PC)	0.80	0.83
	Greedy Thick Thinning (GTT)	0.70	0.75
Three states	Bayesian search (BS)	0.72	0.74
	Peter-Clark (PC)	0.69	0.75
	Greedy Thick Thinning (GTT)	0.72	0.85
Mix states	Bayesian search (BS)	0.77	0.84
	Peter-Clark (PC)	0.78	0.84
	Greedy Thick Thinning (GTT)	**0.83**	**0.85**

It is clear from Table 7 that all nine models demonstrated acceptable levels of predictive accuracy. The mixed-state GTT model had the highest accuracy, the model achieved an 83% accuracy in predicting the actual state of the staff caring factor, and the area under the ROC curve was about 85%. Therefore, the GTT algorithm was selected for the remaining analyses on the mixed-state discretization scheme. Additionally, the literature showed that GTT has been extensively investigated and shown useful in a wide range of application fields, establishing it as a credible choice for creating a data-driven BBN model [46,47]. Table 8 displays the confusion matrix for the staff caring factor, showing the relationship between actual and predicted states. It demonstrates how effectively the BBN model classifies different levels of care and helps assess whether the model consistently predicts outcomes in line with the actual data, providing insight into its reliability for decision-making.

**Table 8:** Confusion matrix for staff caring factor.

Actual	Predicted	
	State 1	State 2
State 1	**34**	10
State 2	11	**67**

**Fig 2:**
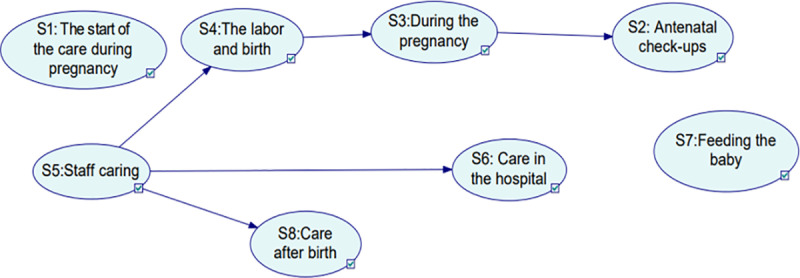
Mixed-state Greedy Thick Thinning network model.

Following the comparison of performance measures, the network was created with the highest performing algorithm, GTT, to explore the statistical dependencies among the various factors and to identify the most critical factors. Therefore, the GTT BBN model shown in Fig 2 was built using the mixed-state dataset.

The BBN model depicted in Fig 2 comprises eight nodes representing the interconnected sections, with 5 arcs demonstrating the dependencies between these sections that can influence overall maternity care. For example, an arc going from node S5 to node S4 indicates that node S5’s probability distribution impacts node S4’s probability distribution.

Based on Fig 2, **S1**: *The start of the care during pregnancy* and **S7**: *Feeding the baby* are not linked to any other factors in the network, this implies that they neither affect the probability of any of the rest of the factors nor their probabilities are affected by the rest of the factors.

The **S2:** *Antenatal check-up* is a child of **S3:** *During the pregnancy.* This suggests that there is probabilistic dependence between them or in other words, the probability of **S3** impacts the probability of **S2**. However, **S3:** *During the pregnancy* is a child of **S4:** *The labour and birth,* indicating that **S3’s** probability is influenced by **S4’s** probability.

**S5:** *Staff caring* is a parent of three **S4**: *The labour and birth*, **S6**: *Care in the hospital*, and **S8**: *Care after birth.* Therefore, the three children’s probability of **S4, S6,** and **S8** are affected by the probability of **S5.**

Moreover, through a series of direct dependencies, indirect dependencies were straightforward to recognize. For example, the following sequence ***S5***→***S4***→***S3***→***S2*** signifies that ***S5****: Staff caring* directly impacts ***S4****: The labour and birth* and indirectly influences ***S3****: During the pregnancy and* ***S2****: Antenatal check-ups*. Notably, with the exception of ***S1****: The start of the care during pregnancy,* ***S5****: Staff caring*, and S7: *Feeding the baby,* all of the factors have a parent. This demonstrates a significant degree of interconnectedness among the eight factors and how they impact the probability of each other.

**Fig 3:**
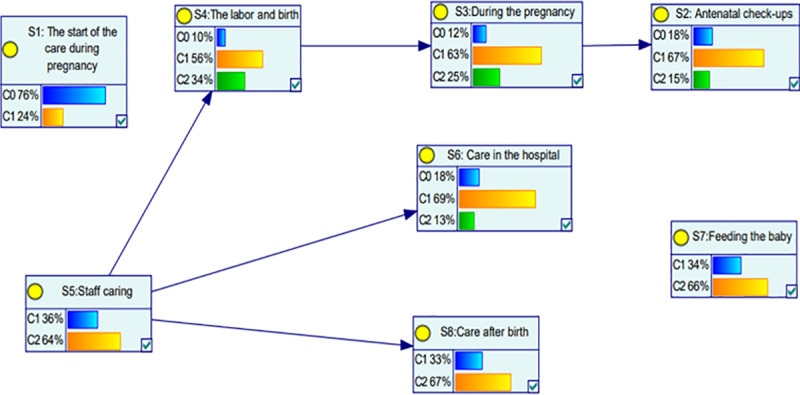
Discrete BBN model representing maternity service factors.

Both the discrete BBN model depicted in Fig 3 and the probability distributions of the eight factors shown in Table 9 reveal that certain factors were discretized into three states, while others were discretized into two states, indicating different levels of experience. For example, “C0” denotes an acceptable experience, “C1” reflects an excellent experience, “C2” reflects an exceptional or beyond excellent experience.

**Table 9:** Probability distribution of the eight factors.

Section	Probability of the patients have an acceptable experience (in percentage)	Probability of the patients have an Excellent experience (in percentage)	The probability of the patients having beyond excellent experience (in percentage)
S1	76	24	
S2	18	67	15
S3	12	63	25
S4	10	56	34
S5		36	64
S6	18	69	13
S7		34	66
S8		33	67

Based on Fig 3 and Table 9, the analysis revealed that **S8: care after birth, S7: feeding the baby, and S5: staff care** had a high probability of patients receiving care rated as beyond excellent (state 3). In contrast, **S1: the start of care during pregnancy** was identified as the least favorable stage, with a maximum rating of 6.72 (on a scale of 10), corresponding to an acceptable level of care (state 1). However, the remaining sections, including **antenatal check-ups and labor and birth,** demonstrated a high likelihood of delivering care that was either excellent (state 2) or beyond excellent (state 3), meeting or exceeding patient expectations.

### 4.3. Sensitivity analysis

**Fig 4:**
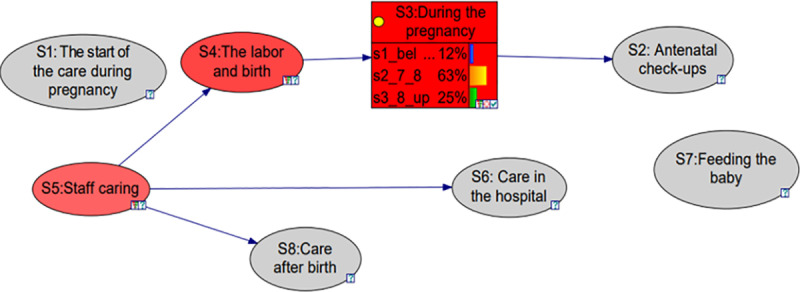
The impact of S3 on the other factors (using sensitivity analysis).

Sensitivity analysis was conducted using two approaches. Initially, I utilized the built-in sensitivity tool within the GeNIe software to examine the relationships between factors. However, this method had limitations, as it primarily captured the unidirectional nature of the interactions. For instance, a change in **S*3****: during pregnancy* directly impacts **S*4****: the labour and birth section* (red highlighted) and has a mild impact on S*5: staff caring* (pink highlighted) as shown in Fig 4. To overcome this, a second approach was conducted manually by individually adjusting the probability of one factor and monitoring its impact on other factors. Specifically, the state2 or state3 probability of each factor was doubled (increased by 100%), while keeping the ratios between the other states unchanged. This manual sensitivity analysis provided a more detailed understanding of the bidirectional interactions, capturing not only the direct relationships but also the indirect effects on factors like S2: antenatal checkups, which were not fully captured by the software’s built-in tool. The detailed impacts of these adjustments are summarized in Table 10. However, both **S1:** and S7: had no impact on the other factors due to the absence of any connections. This approach will enhance the policymakers’ understanding and provide better insight as it is intended to take into consideration both the reverse dependencies and unidirectional dependencies.

**Table 10:** Sensitivity analysis: Association of each factor with the other factors.

The changed factor	The net effect on the other section	The changed factor	The net effect on the other section
S2	**S3 = + 62%**	S5	S2 = + 4%
	S4 = + 30%		S3 = + 7%
	S5 = + 16%		**S4 = + 19%**
	S6 = + 2%		S6 = + 5%
	S8 = + 8%		**S8 = + 16%**
S3	**S2 = + 38%**	S6	S2 = + 2%
	**S4 = + 35%**		S3 = + 5%
	**S5 = + 19%**		S4 = + 12%
	S6 = + 3%		**S5 = + 24%**
	S8 = + 9%		S8 = + 11%
S4	S2 = + 13%	S8	S2 = + 2%
	S3 = + 25%		S3 = + 3%
	**S5 = + 34%**		S4 = + 8%
	S6 = + 5%		**S5 = + 15%**
	S8 = + 15%		S6 = + 2%

***S2****: the antenatal checkups* exhibit a positive influence on all other factors. However, the most significant association was between **S*3****: during pregnancy* and **S*2****: the antennal checks*, this strong association can be because during pregnancy care includes the required antennal checkups [79].

Improving S3: during pregnancy will result in improvements across all other sections. Yet, the most significant improvements are particularly shown in the S2: the antenatal checkups, and S4: the labor and birth.

Positive staff caring can extend their impact to enhance other sections of care and this explains the remarkably high association between **S5:** *staff care* and **S4:** *labour and birth,* and **S8:** *care-after-birth*. Moreover, there is a dependency between **S6**: *the care in the hospital,* and **S5:** *staff care*. This indicates that staff care is the most critical factor among all others.

## 5. Discussion and conclusions

### 5.1. Key findings and contributions of the study

Utilizing the BBN model and following the proposed methodology resulted in five significant findings. Firstly, the NHS Maternity Patient Experience survey had a small sample size and missing values. We used imputation algorithms to create a complete dataset, preserving its integrity and allowing all variables to contribute fully to the BBN model, enabling a more comprehensive analysis. Secondly, among the different discretization approaches examined, the equal-width algorithm stood out.

Thirdly, utilizing the GTT learning algorithm demonstrated superior performance compared to other algorithms, achieving an impressive predictive accuracy of 83%. Fourthly, among the eight factors, there are five interrelationships observed. Notably, **S1:** *The Start of Care in Pregnancy* and **S7:** *feeding the baby* are unique as they remain uninfluenced by any other factors in the network. Both sensitivity and statistical analyses confirmed positive correlations among all factors, indicating that changing the probability of one factor would positively impact others at varying rates. Lastly, the sensitivity analysis conducted offers useful insights into the level of statistical interdependence among the factors. It was revealed that any increase in satisfaction in one factor leads to enhancements in others. Notably, the analysis indicated that **S5:** *staff caring* showed the strongest association with the other factors, and any improvements in other factors were directly related to the quality of staff care. This means that the **S5:** *staff caring* factor is considered the most critical factor influencing the maternity patient’s experience.

This appears to be reasonable as many studies have reported similar findings. For instance, women are more likely to have pleasant maternity experiences when they feel supported, respected, safe, and included in shared decision-making with their healthcare providers [32,80]. On the other hand, disrespectful treatment may result in poor maternity experiences [81]. Moreover, a recent study emphasized that excellent maternity care must be offered with respect [82]. Additionally, it was shown that issues linked to maternal provider behaviors and attitudes offer considerable hurdles to maternity care when compared to other constraints [8].

These studies emphasize the vital role skilled healthcare workers play in delivering high-quality maternal care and improving patient outcomes. A shortage of skilled staff has been shown to adversely impact maternal outcomes and the ability of health systems to maintain readiness [83]. Furthermore, studies suggest that lower nurse workloads and improved work environments result in less missed care and better patient experiences [84]. These findings underscore the critical need to address staffing challenges to enhance both patient satisfaction and clinical outcomes in maternity care.

To translate these insights into practical policies, healthcare providers should focus on strategic workforce planning. This includes initiatives like reducing workloads by setting safe staffing ratios, increasing resources to ensure sufficient supplies and tools, and ensuring an optimal skill mix within healthcare teams. Additionally, better managerial support is needed to foster a positive work environment that encourages compassionate and respectful care.

Notably, the proposed model depicts a relationship between **S*3****: The during-pregnancy* and ***S2****: The antennal check-ups.* The same relationship has been acknowledged in literature. For instance, several studies showed that attending antenatal care and conducting the necessary checkups improved both delivery outcomes and maternal well-being throughout pregnancy [79,85,86].

Besides antenatal care models contributing to preventing adverse consequences during pregnancy, it can play a significant role in ensuring a safe delivery. According to Yohannes et al. (2013), there is a positive association between outstanding antenatal care and women’s satisfaction with safe delivery services in health facilities [87]. This claim is supported by the relationship that is shown in the proposed model between **S4:** *The labour and* birth factor and **S3:** *The during-pregnancy (antenatal care).*

The association of **S5**: *staff caring* with three factors which are **S4:** *The labour and birth*, **S6:** *The care in the hospital*, and **S8:** *The care after birth* seems logical as evidence showed that disrespect and violence during labor can lead to a fear of childbirth, which, in turn, might dissuade women from getting necessary care [88]. Furthermore, according to some studies, hospital care has experienced huge shifts from only considering the sanitary conditions for the patients to putting the focus on the significance of compassion and empathy in healthcare practitioners and their behaviors [89]. Besides, Studies show that the postnatal period is a crucial time for healthcare professionals to be aware of the requirements of new families. Healthcare providers can significantly enhance the whole patient experience by offering support, being attentive, and actively engaging with new parents [90–92].

There has been significant growth in the development and utilization of measures such as surveys, questionnaires, and interviews that are aimed at assessing women’s maternity care experiences in order to improve the quality of care provided [4,93]. However, most studies have focused on isolated phases of maternity care rather than an interconnected process [93]. In contrast, the maternity patient experience survey from England NHS considered all the maternity phases instead. Moreover, to the best of the authors’ knowledge, previous research has not examined the interactions or probabilistic interdependencies among the key factors influencing maternity patients’ experience.

This study addressed this gap by constructing a data-centric BBN model using data from the NHS Maternity Patient Experience Survey, effectively visualizing the interdependencies between several factors that influence maternity patient experiences. Additionally, the incorporation of sensitivity analysis allowed for the identification of the most critical factors impacting patient satisfaction. By quantifying these interdependencies and highlighting the factors that have the most significant influence on patient experience, healthcare managers can prioritize their improvement efforts on areas that will yield the greatest impact.

This knowledge enhances understanding of the maternity care process and supports informed decision-making, ultimately leading to better patient experiences and improved outcomes.

This approach is consistent with previous research that highlighted supportive interactions from care providers can enhance women’s feelings of control and confidence, which are vital for a positive birth experience [22]. Another study links effective person-centered care (PCC) to improved patient satisfaction and health outcomes, particularly when providers are responsive to emotional cues [88]. However, this study goes beyond those findings by modeling the probabilistic interdependencies between key factors, providing deeper insights into how improving one aspect of care can have a positive effect on other areas.

### 5.2. Limitations and future research

While the BBN model provided beneficial insights, it is important to acknowledge the potential limitations. The study’s limitations can be outlined in two primary categories: constraints related to the BBN and GeNIe modeler software, and limitations arising from the dataset used. Firstly, the existing software available for data driven BBN learning is incapable of dealing with continuous variables. Consequently, it becomes necessary to discretize all continuous variables, which may lead to the loss of essential information present in the original data [94].

Secondly, there are certain constraints related to the dataset used in this study. For example, the sample size of 122 observations, although representing trust-level aggregate data, may limit the external validity of the findings. A small sample size can lead to overfitting, where the model captures noise rather than underlying patterns, resulting in high accuracy on the training data but limiting the generalizability to other populations or healthcare settings [95]. While mean imputation is a commonly accepted method for handling missing data, it can introduce bias, especially when data is not missing at random. Replacing missing values with the average of available responses within a section may increase correlations between sections by reducing variability. As this can limit the depth of insights, affecting the model’s accuracy and reliability, we tested different discretization models but further research can also test different imputation approaches. Additionally, a sensitivity analysis on the impact of missing data was not conducted in this study, which might be an important area for future research. Furthermore, while data discretization was used in this study to improve the BBN model, it could also serve as a standalone method to address missing data by preserving the natural sample size. Future research could explore the comparative effectiveness of data discretization and imputation in handling missing values in similar contexts.

Additionally, it is important to highlight that respondents demonstrated a tendency to express exceptionally high levels of satisfaction, possibly influenced by courtesy or gratitude biases. Besides, accounting for the neglected factors in the employed survey, such as women’s expectations, and the importance of the caregiver’s needs and expectations [96] could lead to different rankings of relative importance and variations in interactions among the different factors.

Despite the limitations associated with the NHS data used in this study, the BBN analysis offers valuable insights that can be adapted for practical use in real-world clinical and management settings. The survey content and format, while tailored to this study, can be easily modified to fit different healthcare contexts, enabling healthcare professionals and managers to leverage the results for decision-making and policy development aimed at improving patient care. Future research needs to acknowledge and address the limitations discussed in this study. One potential avenue for improvement is incorporating data from the new NHS maternity patient experience survey from 2022 onwards into the current dataset. This inclusion could lead to deeper insights and increased reliability of the BBN model, ultimately yielding better results. Additionally, considering expert-driven models that incorporate additional factors based on knowledge and experience can provide a more comprehensive view of potentially influential factors, offering valuable support to management and healthcare professionals. Furthermore, it is critical to investigate and evaluate imputation techniques other than the mean imputation utilized in this study to handle missing data. Similarly, different ways of *k*-fold validation might be investigated to examine the model’s performance more thoroughly. Apart from the methodological improvements, future research may also consider conducting a more comprehensive analysis of these relationships to better understand their implications for policy-making. This could enable the formulation of targeted interventions aimed at improving the patient experience in maternal care.

## Supporting information

S1 Appendix Table A1: Complete list of questions for each section. (DOCX)
