# Supplementary material for: Evaluating patient experience in maternity services using a Bayesian belief network model
Source: PLoS One. 2025 Feb 20;20(2):e0318612. doi: 10.1371/journal.pone.0318612 (PMC11841908; doi:10.1371/journal.pone.0318612)
Supplement: S1 Appendix — (DOCX) [file pone.0318612.s001.docx]

Appendix

Table A1 Complete list of questions for each section

| Section | Questions |
| --- | --- |
| S1: The start of the care in pregnancy | - Were you offered a choice about where to have your baby? - At the start of your care in pregnancy, did you feel that you were given enough information about coronavirus restrictions and any implications for your maternity care? - Did you get enough information from either a midwife or doctor to help you decide where to have your baby? |
| S2: Antenatal check-ups | - During your antenatal check-ups, did your midwives or doctor appear to be aware of your medical history? - During your antenatal check-ups, were you given enough time to ask questions or discuss your pregnancy? - During your antenatal check-ups, did your midwives listen to you? - During your antenatal check-ups, did your midwives ask you about your mental health? |
| S3: During the pregnancy | - Were you given enough support for your mental health during your pregnancy? - During your pregnancy, if you contacted a midwifery team, were you given the help you needed? - Thinking about your antenatal care, were you spoken to in a way you could understand? - Thinking about your antenatal care, were you involved in decisions about your care? - During your pregnancy did midwives provide relevant information about feeding your baby? |
| S4: The labour and birth | - At the start of your labour, did you feel that you were given appropriate advice and support when you contacted a midwife or the hospital? - During your labour, did staff help to create a more comfortable atmosphere for you in a way you wanted? - Were you given enough information on induction before you were induced? - Were you involved in the decision to be induced? - Did you have skin to skin contact (baby naked, directly on your chest or tummy) with your baby shortly after the birth? - If your partner or someone else close to you was involved in your care during labour and birth, were they able to be involved as much as they wanted? |
| S5: Staff caring | - Did the staff treating and examining you introduce themselves? - Were you (and / or your partner or a companion) left alone by midwives or doctors at a time when it worried you? - If you raised a concern during labour and birth, did you feel that it was taken seriously? - During labour and birth, were you able to get a member of staff to help you when you needed it? - Thinking about your care during labour and birth, were you spoken to in a way you could understand? - Thinking about your care during labour and birth, were you involved in decisions about your care? - Thinking about your care during labour and birth, were you treated with respect and dignity? - Did you have confidence and trust in the staff caring for you during your labour and birth? - After your baby was born, did you have the opportunity to ask questions about your labour and the birth? |
| S6: Care in the hospital | - On the day you left hospital, was your discharge delayed for any reason? - If you needed attention while you were in hospital after the birth, were you able to get a member of staff to help you when you needed it? - Thinking about the care you received in hospital after the birth of your baby, were you given the information or explanations you needed? - Thinking about the care you received in hospital after the birth of your baby, were you treated with kindness and understanding? - Thinking about your stay in hospital, if your partner or someone else close to you was involved in your care, were they able to stay with you as much as you wanted? - Thinking about your stay in hospital, how clean was the hospital room or ward you were in? |
| S7: Feeding the baby | - Were your decisions about how you wanted to feed your baby respected by midwives? - Did you feel that midwives and other health professionals gave you active support and encouragement about feeding your baby? |
| S8: Care after birth | - Were you given a choice about where your postnatal care would take place? - When you were at home after the birth of your baby, did you have a phone number for a midwifery or health visiting team that you could contact? - If you contacted a midwifery or health visiting team, were you given the help you needed? - Would you have liked to have seen or spoken to a midwife? - Did the midwife or midwifery team that you saw or spoke to appear to be aware of the medical history of you and your baby? - Did you feel that the midwife or midwifery team that you saw or spoke to always listened to you? - Did the midwife or midwifery team that you saw or spoke to take your personal circumstances into account when giving you advice? - Did you have confidence and trust in the midwife or midwifery team you saw or spoke to after going home? - Did a midwife or health visitor ask you about your mental health - Were you given information about any changes you might experience to your mental health after having your baby? - Were you told who you could contact if you needed advice about any changes you might experience to your mental health after the birth? - Were you given information about your own physical recovery after the birth? - In the six weeks after the birth of your baby did you receive help and advice from a midwife or health visitor about feeding your baby? - If, during evenings, nights or weekends, you needed support or advice about feeding your baby, were you able to get this? - In the six weeks after the birth of your baby did you receive help and advice from health professionals about your baby’s health and progress? |
